# A Few Wholesome but Unpalatable Facts

**Published:** 1885-12

**Authors:** 


### CULLINGS FROM CONTEMPORARIES.

### A FEW WHOLESOME BUT UNPALATABLE FACTS.

PROF. E. J. DOERING of Chicago, President of the Alumni Association of Chicago Medical College, in an admirable address before that Society on "The Over-crowding of the Profession," says: "In Chicago *alone* we have more Medical Schools than may be found in the whole Austro-Hungarian Empire, and we graduate in this city this year, more physicians than are usually licensed to practice in the whole German Empire, with a population of forty-five millions!

* * * "Any five physicians actuated by an intense desire to increase a limited practice, can club together, forward $4,50 to the Secretary of State, receive by return mail the necessary charter, and another Medical College is organized. What is true of this State is true of every other State in the Union.

"No wonder, then, that in Chicago alone, we have over 150 Professors and Lecturers on Medicine, or about one professor to every six physicians. * * * In time, the physician who is nothing but a plain M. D. will be, indeed, a rarity—a startling curiosity."

According to this authority, the relative proportion of physicians to population in the several countries is as follows:

| | | | |
|---|---|---|---|
| Sweden, | 1 | to | 7,500 |
| Italy, | 1 | to | 3,500 |
| Germany, | 1 | to | 3,000 |
| Austria, | 1 | to | 2,500 |
| America, | 1 | to | 585 |

And in Chicago, [our estimate from Dr. D's figures] one to every 222 persons.*

Again, Dr. Doering says:

"The action of our diploma-mills, in adding each year, thousands of young men to the ranks of a profession already filled and overflowing, is rapidly producing an army of genteel

*On a basis of 150 Professors—and one to each six Doctors, we would have 900 Doctors, in a population of (estimated) 200,000.—Ed.

paupers, too proud to beg, too honest to steal, but too poor to exist. * * I question *which* is the *greatest* public calamity, an occasional epidemic of cholera, or the regular recurring annual epidemic of some 4,000 doctors let loose on an innocent and unsuspecting public."

Dr. Doering enters an earnest protest against the multiplication of colleges and thus the perpetuation of the great evil; and suggests State Examining Boards as the remedy.

